# Major depression epidemiology from a diathesis-stress conceptualization

**DOI:** 10.1186/1471-244X-13-19

**Published:** 2013-01-11

**Authors:** Scott B Patten

**Affiliations:** 1 Department of Community Health Sciences, Department of Psychiatry & Mathison Centre for Mental Health Research and Education, 4th Floor TRW Building, University of Calgary, 3280 Hospital Drive NW, Calgary, Alberta, T2N 4Z6, Canada

**Keywords:** Mood disorder, Major depressive disorder, Epidemiology, Mathematical models, Simulation

## Abstract

**Background:**

Major depression is a widely used diagnostic category but there is increasing dissatisfaction with its performance. The diathesis-stress model is an alternative approach that does not require the (sometimes arbitrary) imposition of categories onto the spectrum of depressive morbidity. However, application of this model has not been well explored and its consistency with available epidemiologic data is uncertain.

**Methods:**

Simulation provides an opportunity to explore these issues. In this study, a simulation model based on an intuitive representation of diathesis-stress interaction was developed. Both diathesis and stress were represented using continuous distributions, without categorization. A diagnostic threshold was then applied to the simulation output to create nominal categories and to explore their consistency with available information.

**Results:**

An apparently complex epidemiologic pattern emerged from the diathesis-stress interaction when thresholds were applied: incidence was time dependent, recurrence depended on the number of past episodes, baseline symptoms were associated with an increased risk of subsequent episodes and the remission rate declined with increasing episode duration.

**Conclusions:**

A diathesis-stress conceptualization coupled with application of a threshold-based diagnostic definition may explain several of the apparent complexities of major depression epidemiology. Some of these complexities may be artifacts of the nominal diagnostic approach. These observations should encourage an empirical exploration of whether diathesis-stress interactions provide a more parsimonious framework for understanding depression than current approaches.

## Background

Depressive symptoms can be measured using rating scales, which provide an assessment of symptom severity on an ordinal or continuous scale. However, such ratings do not capture important aspects of the concept of a depressive disorder, as this is currently understood. Disorder definitions, and hence most available epidemiologic data, derive from nominal classifications (e.g. major depression (MD) as a named category rather than a scaled rating) that incorporate symptom severity but also thresholds for duration and severity of symptoms and require features such as dysfunction, distress or danger [1]. Diagnostic categories typically play a larger role than symptom ratings in medical practice because they align more closely with clinical decision-making. Since publication of third edition of the Diagnostic and Statistical Manual of Mental Disorders (DSM-III) in 1980 [2], epidemiologic studies have embraced this nominal framework, as have subsequent editions of the manual [1] and the International Classification of Disease [3]. This nominal framework implicitly adopts a particular theoretical orientation towards the epidemiology: an incidence-prevalence-duration framework. In this orientation, there is a “prevalence pool” of depressed people within the population, incidence is an inflow into this pool and the outflow from the pool is due to recovery, remission or mortality. A central aspect of this way of thinking is that the clinical course is characterized by discrete and identifiable transitions between disease states. This framework leads to many familiar epidemiologic concepts (e.g. that the prevalence odds is the product of an incidence rate and mean duration of illness) and is the basis for most disease-modeling approaches. An example of its implementation is the DisMod II program which was until recently used by the Global Burden of Disease project [4].

Categorization of an ordinal or continuous variable into nominal groups can create arbitrary distinctions and runs the risk of obscuring clinically meaningful features. Perhaps in recognition of this, the depression literature has seen the emergence of a complex terminology such as: partial remission [1], residual symptoms [5] and sub-syndromal or sub-threshold episodes [6,7]. Epidemiologically, episodes of the latter type may be difficult to clearly distinguish from non-pathological fluctuations in mood status and adjustment disorders [8].

These difficulties are also evident in the small literature of simulation studies of this condition. All such studies to date have represented the epidemiology from a nominal perspective, employing the general epidemiologic paradigm described above [9-12]. Simulation studies have made an important contribution to the literature about MD epidemiology by indicating, starting with Giuffra and Risch [9] that epidemiologic estimates of lifetime prevalence for MD (which have generally fallen between 10% and 20%) may be underestimates due to recall bias [10,11]. This result has subsequently been confirmed empirically [13,14]. However, the impact of these simulation studies has perhaps been diminished by the complexity of their modeling strategies, e.g. [15].

When viewed through the lens of the general epidemiologic disease model described above, MD epidemiology is indeed complex. For example, there is no simple incidence rate. Incidence appears to decline over time with age [16,17]. Also, there is no single recovery rate. The epidemiologic data indicate that the slope of the cumulative recovery curve for MD episodes is steeper in the early weeks of an episode than in the later weeks [18]. This has required the application of various strategies for modeling such as Markov “tunnels” [19], or the use of lognormal [18] or Weibull [12] distributions to depict a time-varying probability of remission. Various other complexities also emerge, such as the observation that sub-threshold episodes or elevated symptoms occur on a continuum with threshold-defined episodes [20] and are also associated with an increased risk of subsequent major depressive episodes [21]. Finally, the occurrence of depressive episodes in the past predicts their occurrence in the future such that respondents with a large number of recurrences are considered candidates for long-term treatment [22]. Representation of these characteristics, either conceptually in clinical practice or mathematically in a simulation model, involves consideration of complex time-dependent patterns of incidence and recovery and a multiplicity of MD-related health states. However, an interesting possibility is that some of the apparent complexity of MD epidemiology may arise merely as an artifact of forcing a presumably continuous phenomenon into nominal categories.

In order to explore this, it is necessary to initially adopt a non-categorical perspective, treating relevant variables as continuous ones, and then to apply a threshold-based definition to the data. A leading candidate for such a model is the diathesis-stress model, originally formulated (with mixed success) as an interaction between a risk-associated cognitive style and life events, see review by Joiner and Wagner [23], and elsewhere in a social model (including “vulnerability” and “provoking” factors) by Brown and Harris [24]. The concept is now more often applied (although usually without invocation of the “diathesis” and “stress” terminology, e.g. see [25] and [26]) with reference to genetic inheritance, epigenetic modification of gene expression and life events. A depressive diathesis may arise, for example, from multiple genes and from exposure to adversity during development [25]. Neither polygenetic inheritance nor psychosocial adversity fit into “yes” or “no” categories. Some of the depressive diathesis may arise through epigenetic mechanisms due to the ability of early life adversity to reduce (through methylation) expression of a glucocorticoid receptor gene promoter in the hippocampus, producing a longstanding reduction to glucocorticoid-mediated negative feedback inhibition of stress responses [27-29]. Consistent with this idea, adults having a history of childhood adversity show increased reactivity in stressful circumstances and may therefore be at higher risk of MD, see reviews by Taylor et al. [26,30].

The goal of this study was to explore whether a simple diathesis-stress model could reproduce some of the complex patterns typically seen in epidemiologic studies when a nominal diagnostic definition was superimposed on the (continuous) output. An affirmative finding would support, at least in theory, two aspects of the diathesis-stress conception of depressive disorders: (1) its consistency with some of the available epidemiologic data and (2) its parsimony in the sense of its greater simplicity than the incidence-prevalence-mortality framework.

Four aspects of major depression epidemiology that are the focus of the study are: (1) that incidence diminishes over time (with increasing age), (2) that recurrence risk increases with a higher number of past episodes, (3) that elevated symptom levels increase the risk of major depression and (4) that recovery rates decline as the duration of episodes increase.

## Methods

The simulation model used in this study was intended to provide a simple representation of a presumed underlying diathesis-stress interaction producing continuous depressive-symptom output, without attempting to specify whether the diathesis represents genetic, epigenetic, socially or cognitively determined vulnerability. The model was developed in the freely available software NetLogo [31]. In brief, simulated individuals (the model “agents”) were assigned a diathesis value and their exposure to stress was represented by movement of those agents across an environment characterized by different levels of stress. In some of the models presented below (models 2 and 3), values for the stress variable were assigned by random generation from a lognormal distribution with a mean of zero and a user-assigned standard deviation. This resulted in assignment of values for diathesis and stress that were always positive and that had an approximately bell-shaped distribution when the standard deviation was small and was right skewed when the standard deviation was large [32].

**Figure 1:**
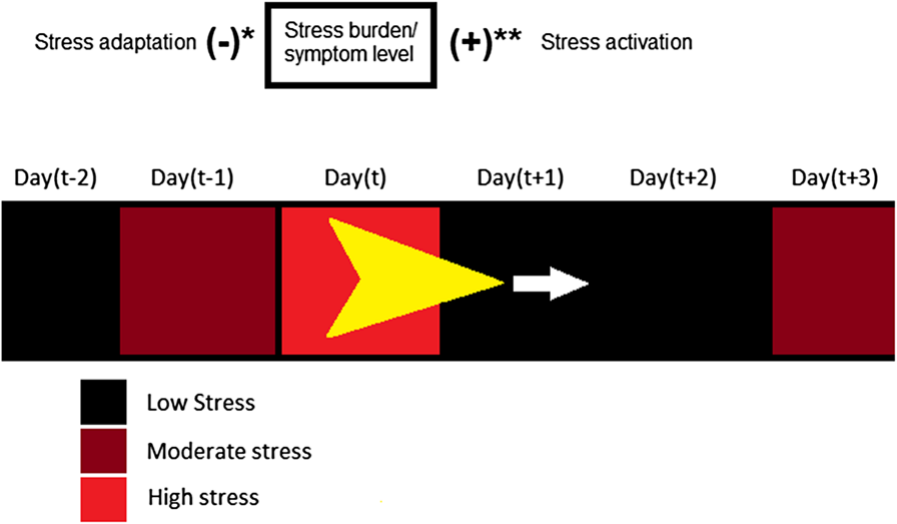
**Schematic depicting an individual (agent) moving over patches.** * see equation 2. ** see equation 1.

Depressive symptom levels were conceptualized as arising from prolonged activation of stress-response systems of the agent. The model used three variables to depict this process: stress activation, stress adaptation and stress burden. The values of each variable were updated in each day of the simulation as the agent moved across the environment, each day coming into contact with an area characterized by a specified stress level. This is depicted schematically in Figure 1, where the yellow polygon is the agent moving over stress regions (shaded squares). In Figure 1 three severities of stress are depicted but this was treated as a continuous variable in the modeling. The burden of stress for a particular agent was determined both by activation and adaptation. On each day activation was an addition to the burden and adaptation as a subtraction from the burden. The equations used to depict activation and adaptation were:

(1) $${\mathrm{Stress}\;\mathrm{activation}}_{\left(t\right)}={\mathrm{stress}}_{\left(t\right)}*\mathrm{diathesis}{\mathrm{Stress}\;\mathrm{adaptation}}_{\left(t\right)}={\mathrm{stress}\;\mathrm{burden}}_{\left(t-1\right)}*$$

(2) $$\left(1/\left(1+\mathrm{adaption}\;\mathrm{constant}*\mathrm{diathesis}\right)\right)$$

Equation 1 represents activation as the product of the entity’s diathesis and the stress level encountered on each day. Equation 2 represents adaptation as the proportion of the total stress burden processed or eliminated in each day. Whereas stress activation was proportional to the diathesis, stress adaptation was inversely proportional. A constant was added to the model in order to allow the influence of diathesis to differ between adaptation and activation. Intuitively, if a depressive diathesis means that vulnerable individuals react more strongly to stressful events (activation) then the diathesis may be expected to primarily affect incidence (see review by Taylor [26]), whereas if the diathesis manifests as a lengthening or persistence of the stress response this may have a greater impact on the duration of symptoms (see for example, epigenetic evidence of impaired glucocorticoid feedback resulting from early life events [28]). The denominator in the right-hand side of equation 2 is formulated such that a value of zero for the adaptation constant would translate into a situation where adaptation to 100% of the stress burden of the preceding day would occur. This constant is labeled an “adaptation constant” in the formulas below and in the models presented in the paper. The change in burden was determined by the difference between stress activation (equation 1) and adaptation (equation 2), as summarized in equation 3 (see also Figure 1):

(3) $$\begin{array}{cc} {\mathrm{Stress}\;\mathrm{burden}}_{\left(t\right)} & ={\mathrm{stress}\;\mathrm{burden}}_{\left(t-1\right)}\\ & +{\mathrm{stressactivation}}_{\left(t\right)}\\ & -{\mathrm{stressadaptation}}_{\left(t\right)} \end{array}$$

Since DSM-IV and ICD-10 definitions of depressive episodes are based on the severity and persistence of symptoms over minimum two week intervals, depressive symptom levels were simulated as a moving average of the current and past 13 days of stress burden, as described above. This is summarized in equation 4:

(4) $$\begin{array}{cc} \mathrm{Depressive}\;\mathrm{symptom}\;\mathrm{levels} & \\ \;=\mathrm{mean}\;\left(\mathrm{stress}\;\mathrm{burden}\;\left(t-13=>t\right)\right) \end{array}$$

**Figure 2:**
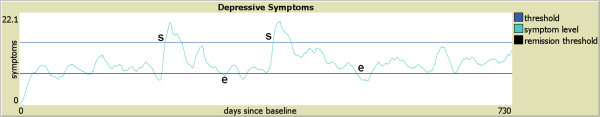
Typical output from model 2* (screen capture). * model parameters: diathesis = 1.4, stress_sd = 0.75, duration constant = 3, threshold = 15, remission stringency = 0.5. s = start of episode. e = end of episode.

Finally, the simulation model included a threshold value and a remission stringency value. Entities were classified as entering an episode whenever their symptoms exceeded the threshold value. A depressive episode is clinically usually not considered to be over until symptoms are well below the diagnostic threshold (the concept of remission) [33]. Remission stringency specifies the extent of symptomatic improvement required before the episode can be considered over. For example, if the remission stringency value is 0.5 symptoms must fall to less than 50% of the diagnostic threshold value before the episode is considered over. The relationship between these thresholds and episode duration is presented schematically in Figure 2. It should be emphasized that the threshold and remission stringency variables are not intrinsic to the diathesis-stress interaction that the model represents. Rather, these are variables used to process the output data as if it were epidemiologic data with analogues of threshold and remission-based nominal definitions applied to it. Additional file 1 presents a more structured description of the model using a table format adapted from Railsback and Grimm [34].

**Figure 3:**
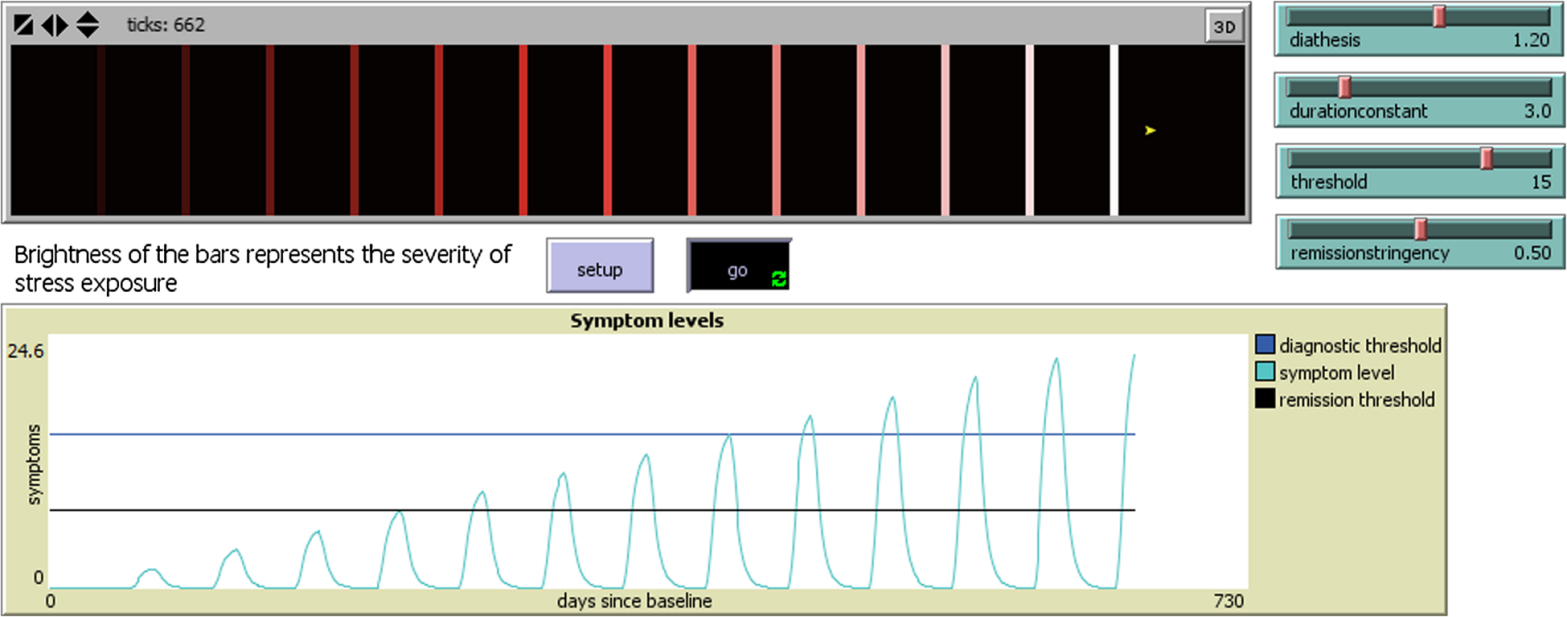
A screen capture from model 1*, near the end of a simulation. * See http://people.ucalgary.ca/~patten/model_1.html.

The amount of time that an agent spends with symptoms exceeding the diagnostic threshold before dropping below the remission threshold was the model’s representation of the duration of a nominally classified episode. To illustrate the model, an applet is available through this reference [35]. The model is labeled Model 1. As the entities pass through the stressful intervals depicted by the bars their level of symptoms increases and then subsequently decreases after they pass through the bars into a low stress zone. A screen capture of the applet output is presented in Figure 3.

A second version of this model (Model 2) provides output at the level of an individual agent, simulating the experience of this agent over 2 years (730 days). Again, this is a Java applet and can be examined using a web browser using a link provided in the reference list [36]. In this model, the distribution of the stress variable is assigned randomly to the environment over which the agent passes. The parameter values for the stress distribution can be changed by the user. Note that the button for the stress variable is labeled “stress_sd” because the distribution of stress is represented using the logarithm of a normal distribution with a mean of zero and this value for the standard deviation of the normal distribution. Note that when the model is initialized, the placement of the agent on the y-axis of the output window is random, so that the pattern of exposure to stress is not the same for each simulated agent even if the model parameters are not changed. Running the model several times using the same set parameters may result in episodes occurring in some simulations but not others.

Model 3 builds from the previous ones by randomly assigning diathesis values to each agent, simulating the experience of a series of agents (the number of which can be changed) and calculating output parameters: the incidence proportion (over the specified interval), the mean first episode duration and the mean episode count over the specified simulation interval. This model demonstrates how inter-individual variation in diathesis can be added to the prior model in order to simulate a population sample. Model 3 can be accessed through this reference [37].

The model that was used to produce the simulated output for data analysis in this study is attached as Additional file 2. This model is the same as Model 3, above, except that the simulation interval is set by default to 10 years (3652 days), the output window on the interface is expanded to accommodate this, and some additional outcome variables are recorded by the model in a data file. For example, the model output records the number of episodes occurring in the first nine years of the simulation in order to evaluate the emergence one of the complexities under investigation: whether the number of episodes in the first nine years would predict whether an episode would occur in the 10^th^ year. A set of parameters was calibrated by trial and error to produce roughly the expected pattern of 10 year incidence (about 13%) [14], mean episode counts over 10 years (between 2 and 3 episodes for each agent having an episode) [12] and a mean first episode duration of about 11 months. This one-year episode duration may seem long for MD, e.g. see [38], but the mean duration of MD episodes have been found to be strongly influenced by a small number of very long episodes such that the distribution of episode durations from the simulation model does follow the familiar pattern, e.g. see [18], with this parameterization. The settings used in the simulation were 0.20 for the diathesis standard deviation, 0.75 for the stress standard deviation, 15 for the threshold value, 0.5 for the remission stringency and 3.0 for the adaptation constant. A trial simulation of 100,000 individuals with this model resulted in: a 15% cumulative incidence, mean episode count (among those with and episode) of 2.8 and a 314 day mean episode duration.

These parameters were then used to simulate 100,000 observations, creating a dataset with which to evaluate the specified epidemiologic patterns. This dataset, in the form of a comma separated values file (Additional file 3), was imported into the statistical software Stata [39] for analysis. Additional file 4 includes the same dataset in Stata format. A Stata “do” file in Additional file 5 was used to generate the results of the study, as reported below.

Additional information on characteristics of the model may be found in Additional file 6. This document presents a series of simulations, examining the effects of altering model parameters on two of the epidemiologic outputs: incidence and mean duration of the first episode.

## Results

**Figure 4:**
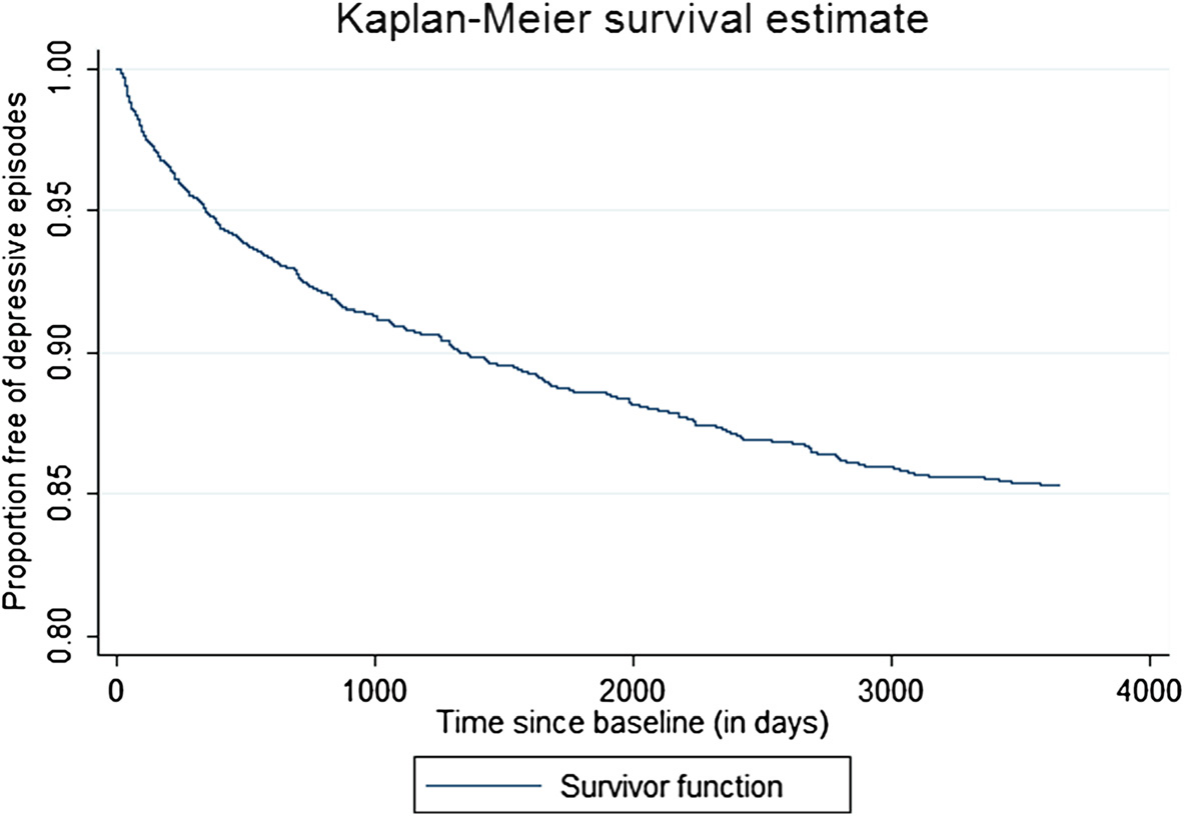
Incidence* over 10 years of a 3652 day simulation run. * incidence is the slope of the Kaplan-Meier curve.

The first question addressed was whether simulated incidence would decline over time. Figure 4 presents a Kaplan-Meier curve for the onset of episodes. This analysis excludes simulated individuals already depressed at baseline (day 14, the first day when the moving average could be calculated). The slope of the curve flattens from left to right, indicating declining incidence.

**Figure 5:**
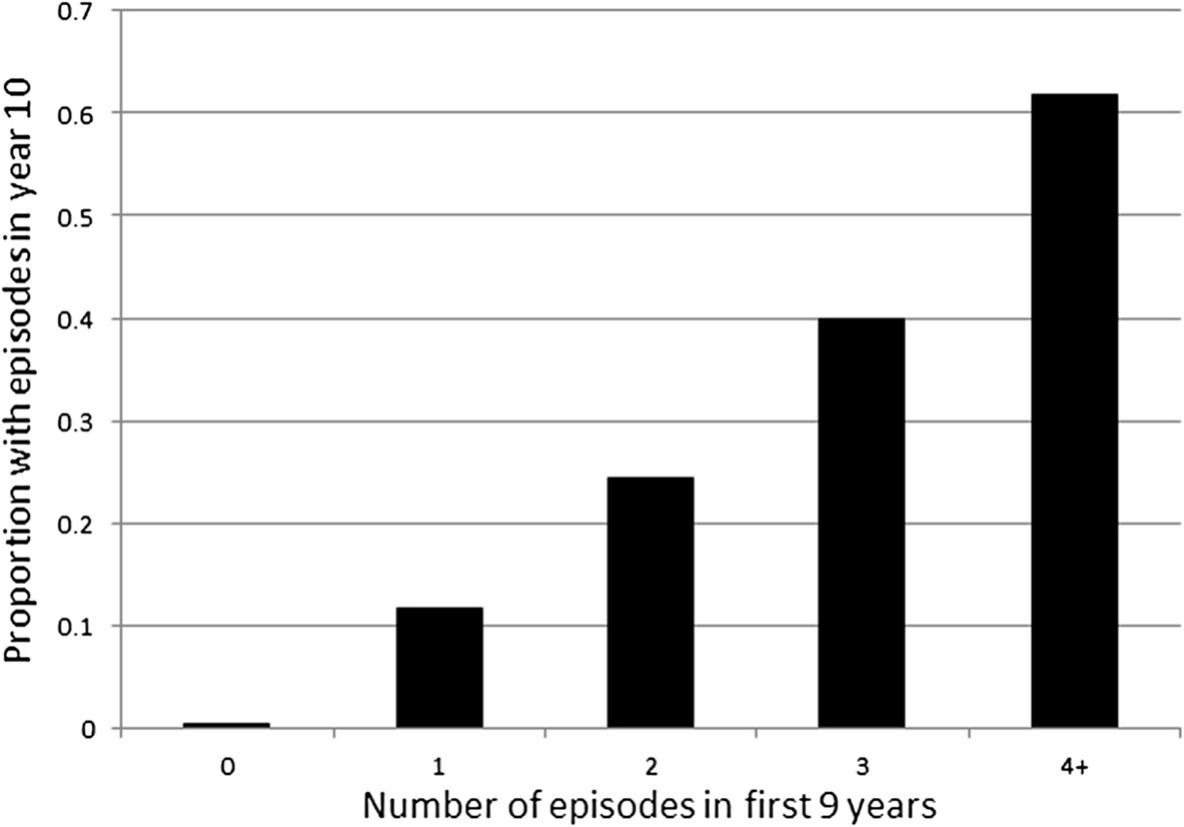
Recurrence in year 10, by number of episodes in years 1–9.

The second question to be addressed was whether recurrence risk increases with a larger number of past episodes. This was addressed by tabulating episode incidence in the final year of the 10 year simulation interval according to how many episodes had been experienced during the first nine years. There is a dramatic association between the number of episodes in the earlier interval and the risk of having one in the final year of the simulation, see Figure 5.

**Figure 6:**
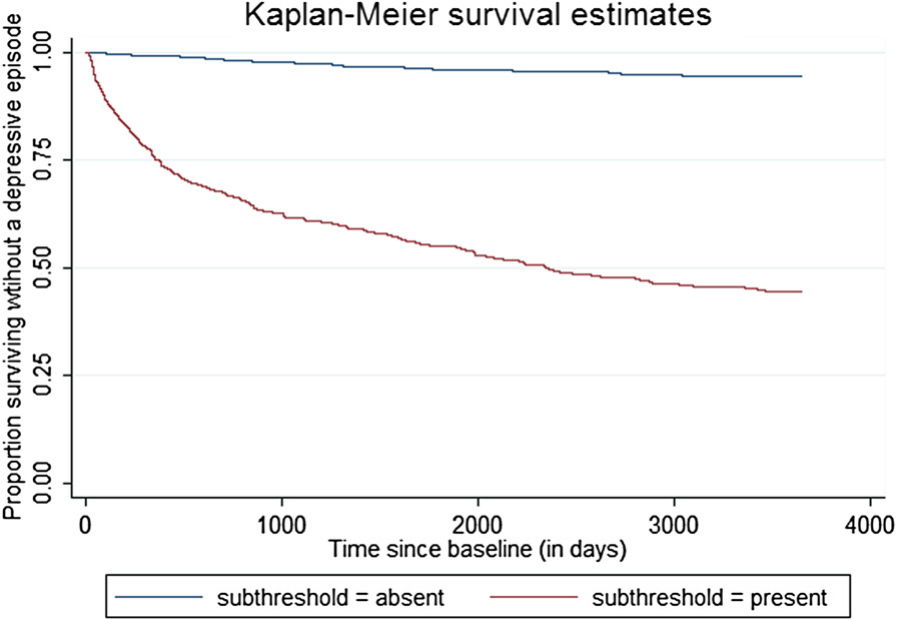
Incidence over 10 years, by elevated* depressive symptoms at baseline. * scores > 6 (approximately 50% of the diagnostic threshold) were considered elevated.

The third question was whether depressive symptoms at baseline or sub-threshold episodes at a baseline time point would predict subsequent incidence. To assess this, baseline scores were calculated at the first possible time (day 14 in the simulation) and Kaplan-Meier curves were generated for both groups. Respondents exceeding the diagnostic threshold value at the baseline time point (depressive symptoms at baseline were calculated at day 14) were excluded from this analysis. Score greater than 6 (approximately half the diagnostic threshold value in the simulations presented) was taken to indicate elevated symptoms. Those with higher baseline symptoms had a much higher subsequent risk, see Figure 6.

**Figure 7:**
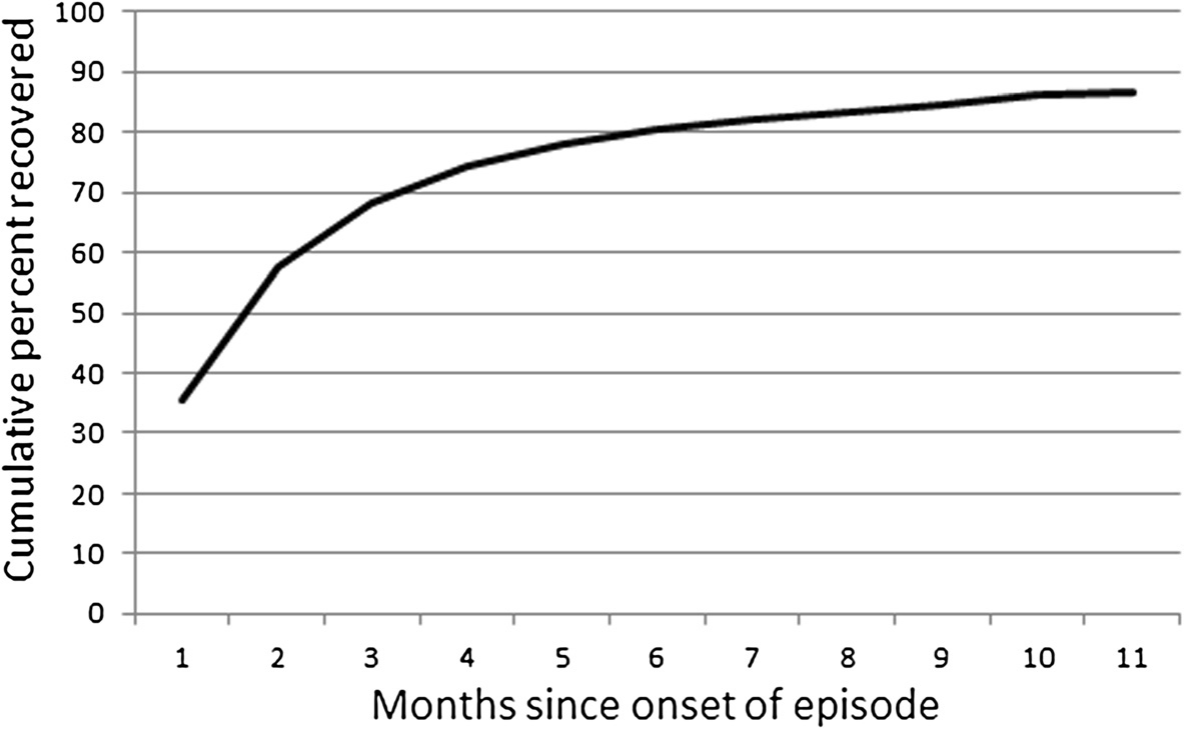
Simulated cumulative recovery from MD episodes, by month.

The fourth question of interest was whether the probability of recovery would diminish with increasing episode duration. Figure 7 shows the cumulative probability of recovery among first episodes as a function of time. The rate of recovery is high in the early months and then diminishes subsequently, as is typically reported for major depression [18]. A fairly large proportion (13%) of the simulated cohort had not recovered after one year.

## Discussion

The general idea of a diathesis-stress model is intuitively appealing and not so complex as to be inaccessible to intuition. The simple formulas that comprise this simulation model merely reflect a simplified, intuitive representation of a diathesis-stress interaction. What these models demonstrate is that this simple and basic representation of an intuitive idea produces resemblances of epidemiologic data concerning depressive disorders. As such, the importance of this study is its tentative identification of the diathesis-stress model as a promising alternative framework for understanding these disorders. Given mounting awareness of the limitations of the current approach to diagnosis [40,41] such tentative exploration of alternative perspectives seem warranted. These results should encourage further exploration of the diathesis-stress relationships that may exist in depressed patients.

Major depression has been viewed as an episodic condition with a complex epidemiology characterized by age, time and state-dependent rates of transition, with afflicted persons moving in and out of episodes according to these complicated dynamics. Through the lens of a diathesis-stress approach it is also possible to regard this condition as representing interplay between a personal vulnerability (diathesis) to the effects of stress and exposure to stressful life events. Diathesis-stress interactions are expected to result in fluctuating levels of symptoms as individuals with various degrees of vulnerability encounter various levels of stress in their lives. These models show that application of a threshold to a fluctuating level of symptoms can produce the appearance of an episodic disease course, at least in a subset of the population whose diathesis-stress interactions leave them neither always below (never depressed) nor always above (chronic depression) diagnostic thresholds. Whereas major depression has been viewed as an inherently episodic condition, the results presented here are consistent with the possibility that diathesis-stress dynamics may be more fundamentally important to the depressive disorders.

The tendency of the number of prior episodes to predict subsequent recurrence (see question 2, above) might be taken as evidence of kindling [42] or “scar” [43-46] related phenomena, but the simulations presented here suggest another possibility: that this and other features may occur as an artifact of applying a threshold-based definition to a fundamentally continuous underlying process. Of note, the use of the term “scar” has recently been invoked to describe heightened depressive diatheses arising from early life adversity [47], a usage differing from the traditional concept of a “scar effect” (an effect of a depressive episode that persists after remission is obtained). This non-traditional usage fits nicely with the conceptualization modeled here. At a physiological level, the diathesis depicted in this study using a probability distribution might partially represent “limbic scars” arising from early life adversity, manifest along a continuum. Among other possible explanations, relevant changes may be related to polygenetic risk [25] or to adversity-induced methylation of promoter regions for hippocampal expression of glucocorticoid receptors – leading to increased stress sensitivity and diminished stress adaptation [48].

The model presented here is not intended to accurately reflect any real-world phenomena or to be used as a decision-support or prediction tool. The model has a conceptual rather than empirical basis. Furthermore, even conceptually, the model does not attempt to account for several potentially important factors. For example, whereas stress has been modeled as interacting passively with a diathesis, in reality people learn from their experiences and can also learn skills to cope with stressful events. The probability distributions selected for use in the model are arbitrary and were selected merely because they seemed to “make sense” intuitively. Conceivably, different patterns of exposure to stress could, in themselves, produce an episodic pattern, even without individual variation in vulnerability (diathesis). Despite these concerns, these results encourage the idea that empirical confirmation of diathesis-stress interactions (a rapidly developing area within the epigenetics literature [48]) may lead to simpler and more useful ways of understanding these conditions than the current approach has provided.

There are additional limitations to this study. Episode duration was examined only for the first episode, as this was the simplest to record from the model. In principle, episode duration (rather than specifically first episode durations) was the targeted output, so the strategy that was adopted in capturing the output is a limitation in this respect. Also, some of the agent’s variables could have been treated as global variables in the models since each models only a single individual at a time. More sophisticated agent-based simulation approaches, better informed by the rapidly developing biological literature, will play a role in advancing knowledge about these disorders.

## Conclusions

Whereas major depression is usually characterized as an episodic condition with a very complex epidemiology, it may instead (at least in some cases) represent a much simpler pattern of environmental interaction with heightened vulnerability to stress. Some of the complexity associated with the epidemiology of this condition may arise as an artifact of applying threshold-based diagnostic definitions to an inherently dimensional underlying process. The diathesis-stress concept is a strong candidate framework to supersede the currently dominant threshold-based nominal approach.

## Competing interests

The author(s) declare that they have no competing interests.

## Author’s contributions

Dr. P was responsible for all aspects of the study.

## Pre-publication history

The pre-publication history for this paper can be accessed here:

http://www.biomedcentral.com/1471-244X/13/19/prepub

## Supplementary Material

Additional file 1 Description of the Simulation Model. Click here for file

Additional file 2 The model used to produce simulated output for the reported data analyses. Click here for file

Additional file 3 Simulation output, in comma-separated format. Click here for file

Additional file 4 Simulation output, in comma-separated format. Click here for file

Additional file 5 The Stata ‘do’ file used to produce the reported estimates from the simulation output. Click here for file

Additional file 6 **Model output: Effect of model parameters on incidence and first episode duration.** (PDF 297 kb) Click here for file
