# Supplementary material for: Major depression epidemiology from a diathesis-stress conceptualization
Source: BMC Psychiatry. 2013 Jan 11;13:19. doi: 10.1186/1471-244X-13-19 (PMC3549292; doi:10.1186/1471-244X-13-19)
Supplement: Additional file 1 — Description of the Simulation Model. [file 1471-244X-13-19-S1.pdf]

Additional File 1. Description of the Simulation Model*

Overview	1. Purpose	The purpose of this model is to explore the possibility that some of the complexities of major depression epidemiology may arise as an artifact of the way in which this condition is diagnosed. This is important since biological research has not (yet) been able to define depressive disorders as a distinct state apart from a continuum of depressive symptom severity. The diagnosis (of major depressive episode, for example) is made by application of a threshold-based definition to a continuous underlying variable: depressive symptom levels. Of course, the concept of a diagnosis also implies other aspects than symptom severity (e.g. in DSM-IV, the general definition of a mental disorder incorporates concepts of associated dysfunction and elements of danger to self or others). Complexities that have been reported in the epidemiologic literature about major depression (as this condition is currently defined) may result from actual features of an inherently episodic condition, or as artifacts of application of a threshold to a fluctuating symptom level. Such complexities include: incidence that declines with age, remission rates that decline with increasing chronicity, and the ability of past episodes and current symptoms to predict future episodes.<br><br>The exploration was accomplished by developing a model of depressive symptoms in which those symptoms are represented as a continuous outcome. This model was based on a diathesis-stress conception, in which interactions between individuals with varying levels of vulnerability (diathesis) to environmental (i.e. biological, psychological and social) stressors. Next, continuous data from this model were captured and a diagnostic threshold is applied to categorize the output nominally: depressed or not. The duration of episodes is also recorded. The data are then exported to a statistical analysis program and analyzed as epidemiologic data.
	2. Entities, state variables, and scales	The simulated entities represent people. These are tracked by the model over a 10 year period. Depressive symptoms are conceptualized as a moving average of stress-response system activation over 14 days (the minimum period for a depressive episode in the major diagnostic classifications).<br><br>Each entity is assigned a value for diathesis – or vulnerability to increasing depressive symptoms. The values assumed by this variable are assigned randomly by taking the inverse logarithm of a normal distribution with mean = 0 and standard deviation specified by the user of the model (see model 3). Please note that models 1 and 2 in the paper are presented to illustrate the way in which the model works, but do not incorporate the idea of variable levels of diathesis between people in the population – a characteristic that is represented in model 3. The resulting distribution can be approximately bell-shaped or highly skewed depending on the standard deviation selected, but the diathesis variable always has a positive value.<br><br>As the model entities move through time they move over a conceptual space characterized by varying levels of stress. In the NetLogo software, the conceptual space consists of "patches" and the level of stress is represented using a variable assigned to those patches. The values of this variable are assigned randomly by taking the inverse logarithm of a randomly generated normally distributed variable with mean zero and a standard deviation assigned by the user (see models 2 and 3).<br><br>An entity can be in two states: when their depressive symptoms are above a threshold they are always considered depressed. When they are below the threshold they are generally non-depressed. However, remission from a depressive episode does not occur until a person's symptoms drop below a level that is less than the symptom threshold for diagnosis, e.g. 50% of this threshold value. For this reason, the models include a "remissionstringency" variable that defines the proportion of the threshold value to which a depressed entity's score must drop to in order for that entity to be regarded as being in remission. If "remissionstringency" is 1.0, then an entity enters remission as soon as they drop below the threshold value, if it is 0.5 then they would enter remission at 50% of the threshold value. The latter value is more consistent with the concept of remission as this is applied in clinical practice and epidemiologic research.<br><br>The model records the time of onset of episodes and when the previously depressed entities enter remission. The time difference between these two values is the duration of an episode. Duration of the first episode is recorded as a variable attached to the individual people simulated by the model.
	3. Process overview	The level of symptoms at a given point of time is denoted as a burden of stress ("stressburden") and is related both to activation of a simulated stress response system and adaptation of that system to stress. Stress activation is defined as a product of stress level (as assigned to the patch over which an entity is situated) and the diathesis variable possessed by the entity. There is also a process of stress adaptation, such that a proportion of the effect of stress is removed at each day of the simulation (depicted in NetLogo as a "tick"). The stress burden on any given day is the burden carried over from the previous day plus new stress activation minus stress adaptation, see equations 1 and 2:<br><br><u>Equation 1</u>:<br>$\text{Stress activation}_{(t)} = \text{stress}(t) * \text{diathesis}$<br><u>Equation 2</u>:<br>$\text{Stress adaptation}_{(t)} = \text{stress burden}_{(t-1)} * (1/(1 + \text{adaptation constant} * \text{diathesis}))$<br><br>Where the "adaptationconstant" reflects the idea that adaptation to stress is inversely proportional to a person's level of vulnerability (diathesis) but not necessarily with exactly with the same proportionality as the effect of a diathesis on stress activation.<br><br>As noted above, the level of burden is assumed to be reflected in an entity's simulated emotional state on a particular day. Depression is taken to refer to a more sustained change in mood, and is therefore incorporated into the model as a 14 day running average of these daily levels of stress-response system activation. Persistent elevation of symptoms is taken as a representation of depression, and persistent elevation that exceeds a diagnostic threshold is taken as a representation of an episode. The change in burden was determined by the difference between stress activation (equation 1) and adaptation (equation 2), as summarized in equation 3:<br><br><u>Equation 3</u>:<br>$\text{Stress burden}_{(t)} = \text{stress burden}_{(t-1)} + \text{stressactivation}_{(t)} - \text{stressadaptation}_{(t)}$<br><br>Since DSM-IV and ICD-10 definitions of depressive episodes are based on the severity and persistence of symptoms over minimum two week intervals, depressive symptom levels were simulated as a moving average of the current and past 13 days of stress burden, as described above. This is summarized in equation 4:<br><br><u>Equation 4</u>:<br>$\text{Depressive symptom levels} = \text{mean (stress burden (t-13=>t))}$
Design Concepts	4a. Basic principles	The formula above calculates a level of depression as an entity encounters variable levels of stress over time. The model can simulate the experience of a single entity (models 1 & 2), or of a population of entities (model 3). Population variability in depressive diathesis is represented in the latter instance by random assignment of a value for this variable to successively simulated entities (model 3), see #2 under "Overview" above.
	4b. Emergence	In order to explore whether the three noted complexities of depression epidemiology can arise as an artifact of defining depressive episodes as nominal characteristics through application of a threshold for simulated depression severity, the simulation output is collected into a data file that can be subjected to typical epidemiologic analysis techniques to see whether the complex patterns emerge.
	4c. Stochasticity	The patches in the model over which the entities pass at a rate of one patch per day are each randomly assigned a stress value, as described above. The patches are arranged (in models 2 and 3) in a rectangular array, as depicted on the models interface. This means that the patterns of stress encountered by different entities in successive simulations (model 3) vary. The entities all start at the y-axis of the NetLogo interface (time = 0 days). Their position on the axis is assigned randomly so that there is variation in the experience of stress when the experience of multiple entities is simulated (model 3).
Details	5. Initializatio n	At initialization, stress values are assigned to all patches in models 2 & 3. In model 1, stress values are assigned to bars in order to simplify the interface for purposes of illustration. A value for diathesis is assigned to the first entity to be simulated in models 1, 2 and 3. In model 3 (which depicts multiple entities in a population) the diathesis values are randomly assigned from the specified (see above) distribution for each new entity. The experience of each new entity is simulated separately and successively.
	6. Input variables	These are described above, and are listed here as they appear in the interface of the population (model 3, as opposed to individual entity simulations in models 1 & 2): (1) the standard deviations of the normal distribution from which the diathesis and stress variables are generated, (2) the symptom severity threshold that defines a depressive episode when it is characteristics of a two-week period, (3) the stringency of the definition to identify remissions and (4) the proportionality constant for calibrating the rate of stress adaptation.

* Adapted from Railsback and Grimm. Agent-based and individual-based modeling. A practical introduction. Princeton: Princeton University Press, 2012.
