# Supplementary material for: Major depression epidemiology from a diathesis-stress conceptualization
Source: BMC Psychiatry. 2013 Jan 11;13:19. doi: 10.1186/1471-244X-13-19 (PMC3549292; doi:10.1186/1471-244X-13-19)

Additional file 6: Model Output

Effect of model parameters on incidence and first episode duration

Contents

1. The stress_sd parameter. 2
2. The diathesis_sd parameter. 3
3. The threshold parameter 4
4. The duration constant parameter 5
5. The remission stringency parameter 6

1. The stress_sd parameter.

The stress_sd variable introduces variability and a right skew to the distribution of stress levels encountered by the agents. At higher levels of stress_sd agents will be exposed to increasingly high levels of stress. This leads to a higher incidence of major depression, but since episodes occur in the absence of a strong diathesis – the duration of the episodes decreases with increasing values.

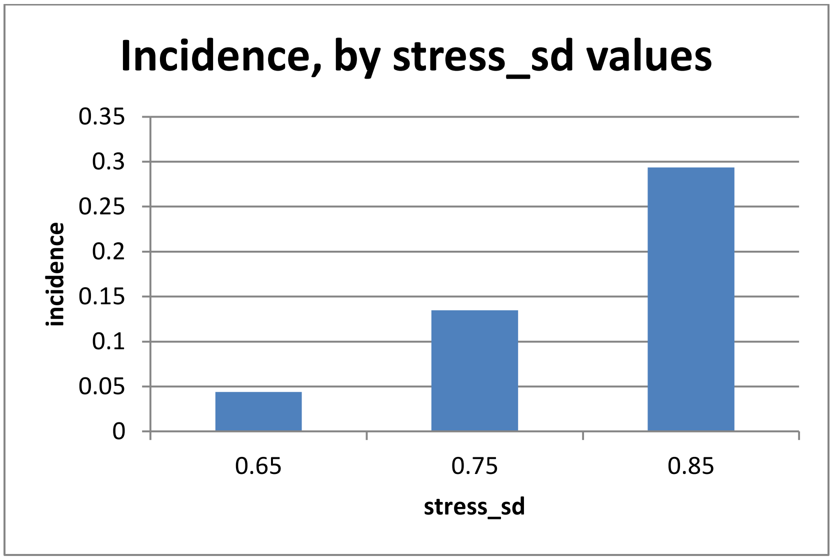

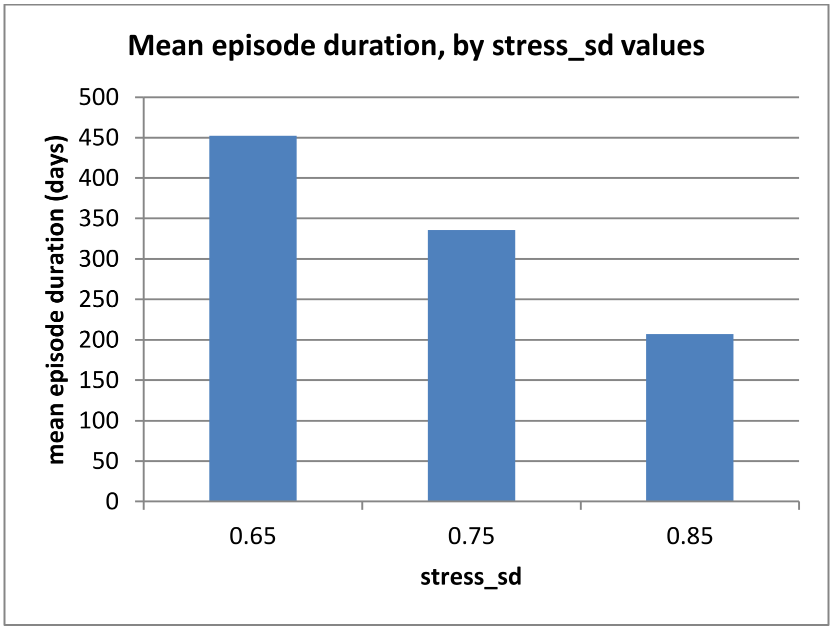

2. The diathesis_sd parameter.

Increasing values of this parameter lead to higher values for the diathesis for some agents, and introduce right skew. The result is a simulated population containing some members with a strong diathesis. Increasing values lead to higher incidence and longer episode duration when an episode occurs.

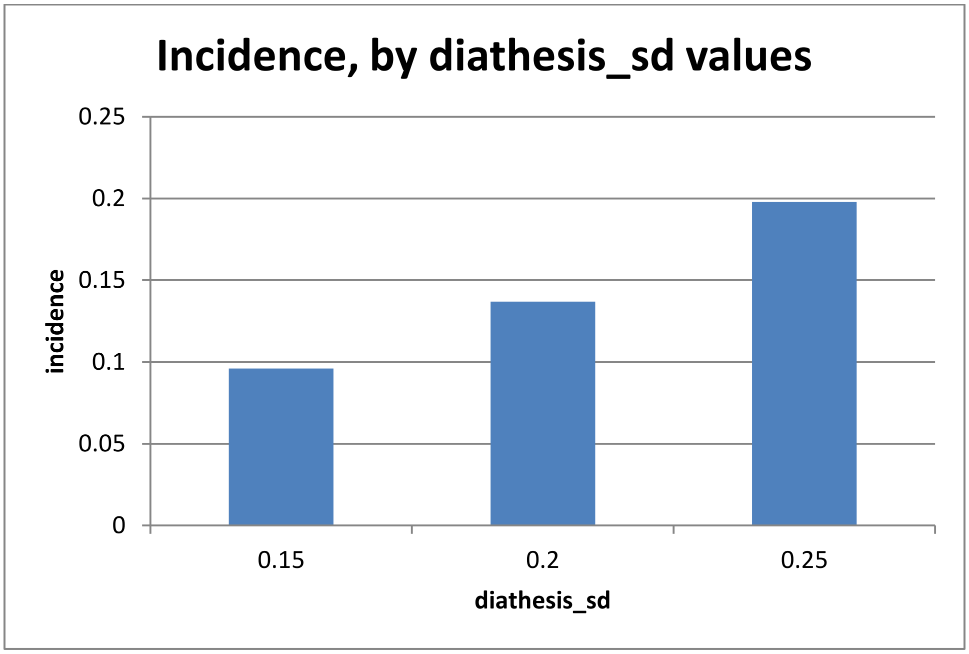

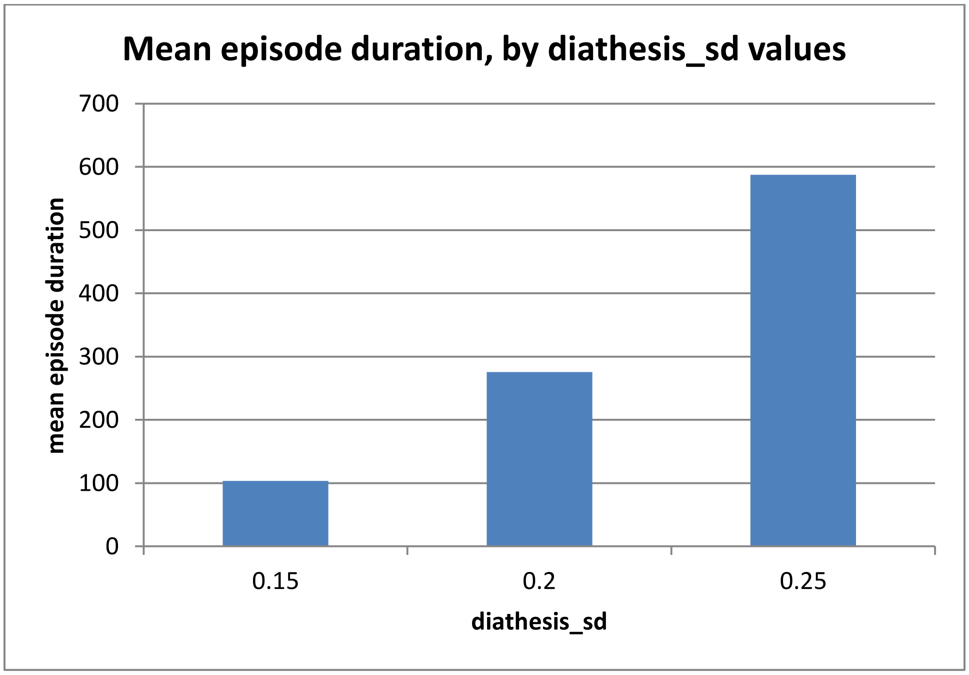

3. The threshold parameter

A lower threshold leads to higher incidence and higher episode duration, the latter because the threshold is consistently exceeded by a proportion of the simulated population when the threshold value is low.

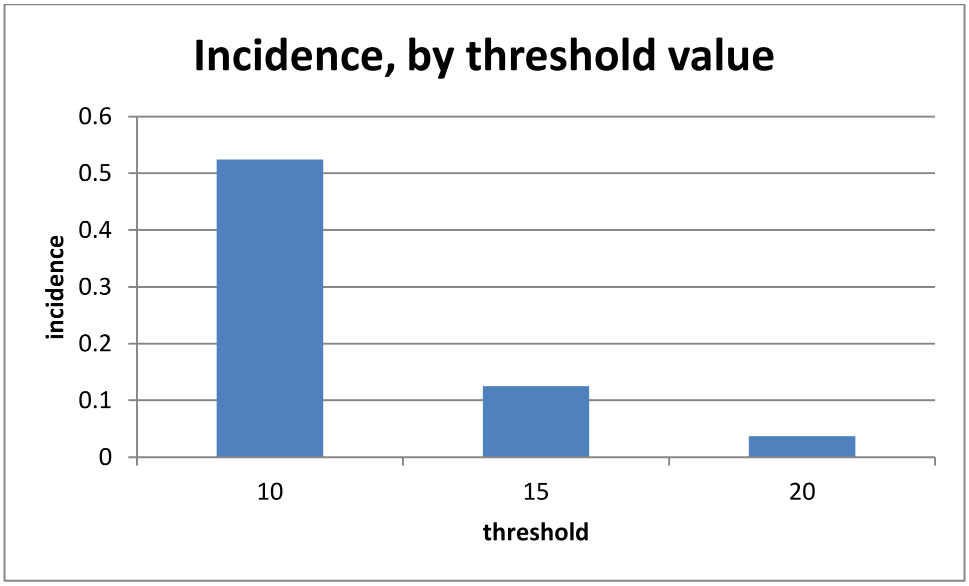

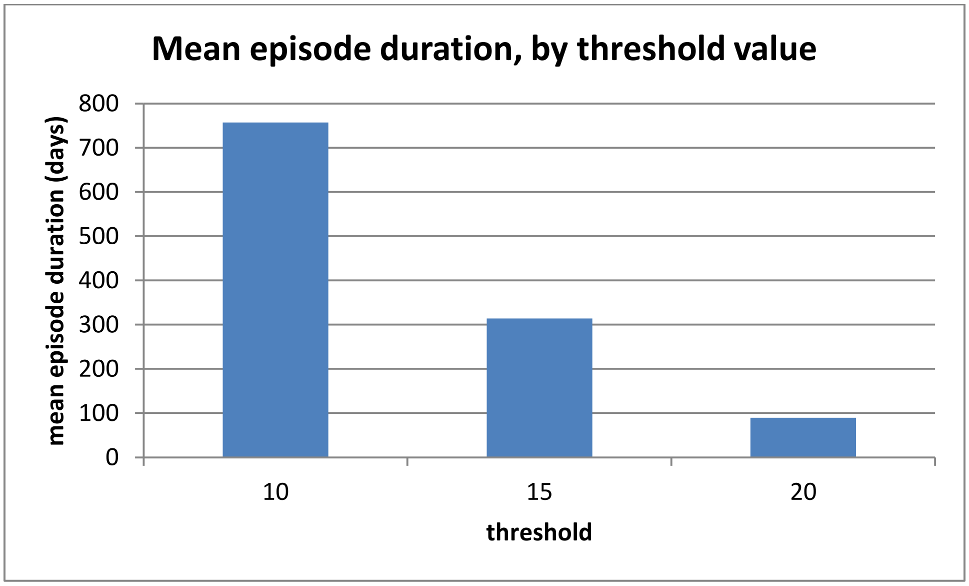

4. The duration constant parameter

The duration constant slows the process of adaptation, as depicted in the model. It has a strong effect on simulated episode duration. There is also a strong effect on incidence, because slow rates of adaptation allow the accumulation of stress burden.

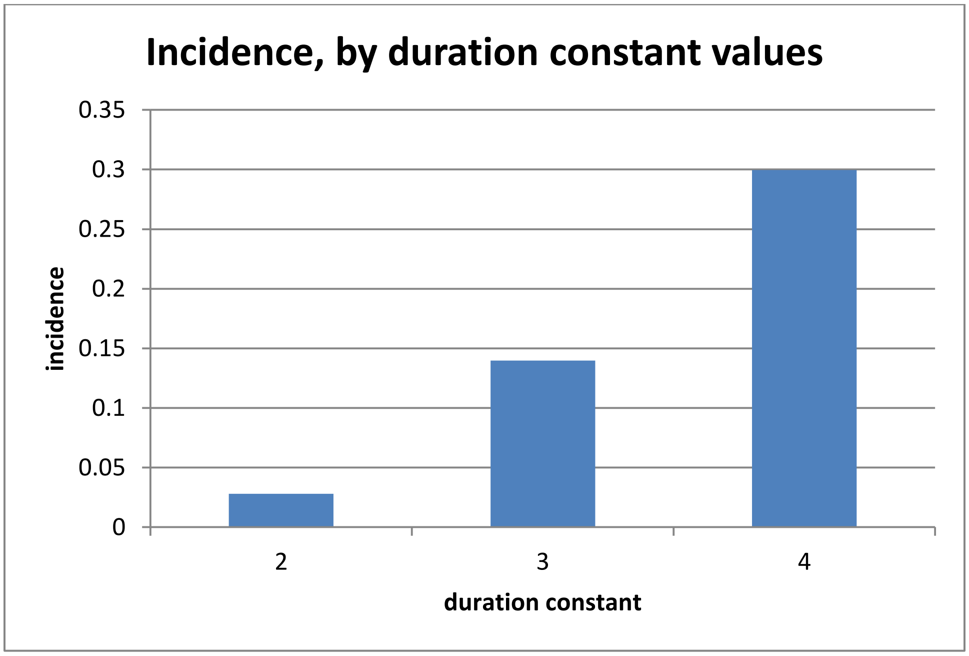

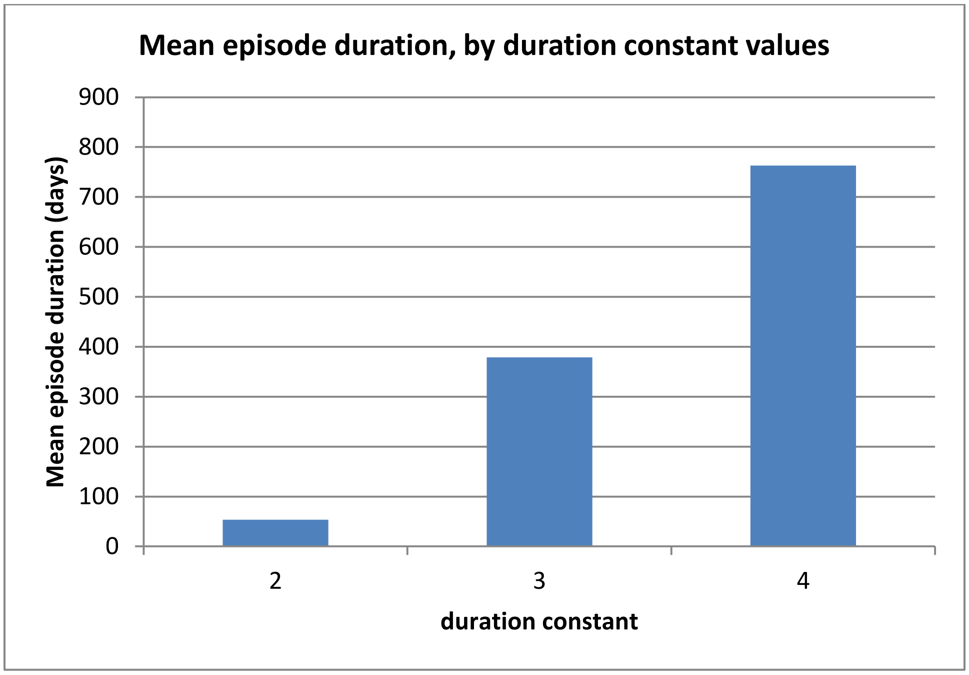

5. The remission stringency parameter

The remission stringency parameter does not have a large impact on incidence, but has a large impact on mean episode duration. Very stringent definitions of remission prolong the apparent duration of episodes.

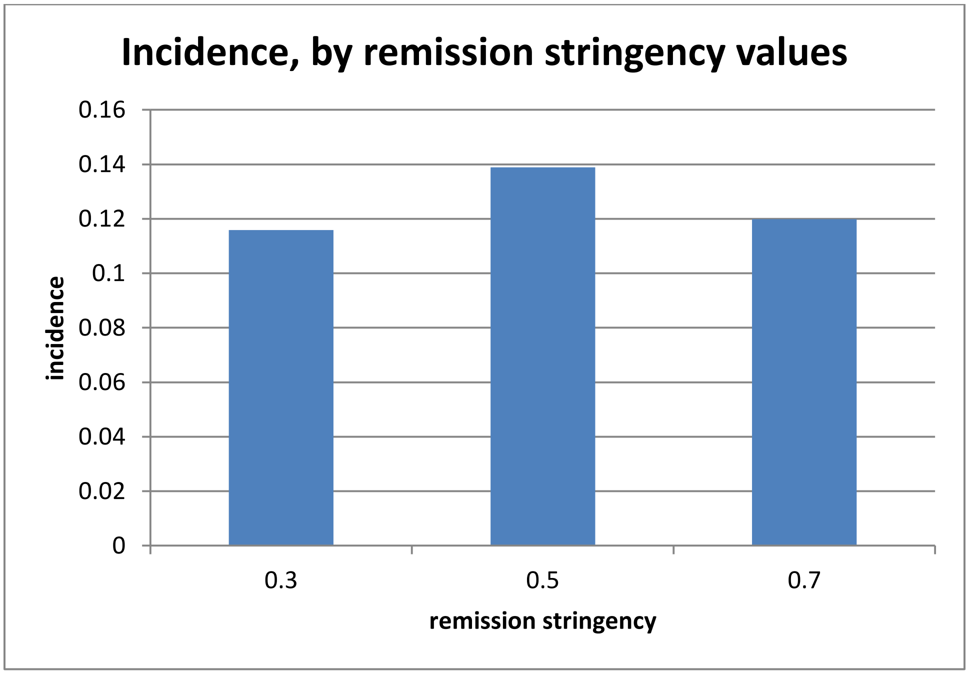

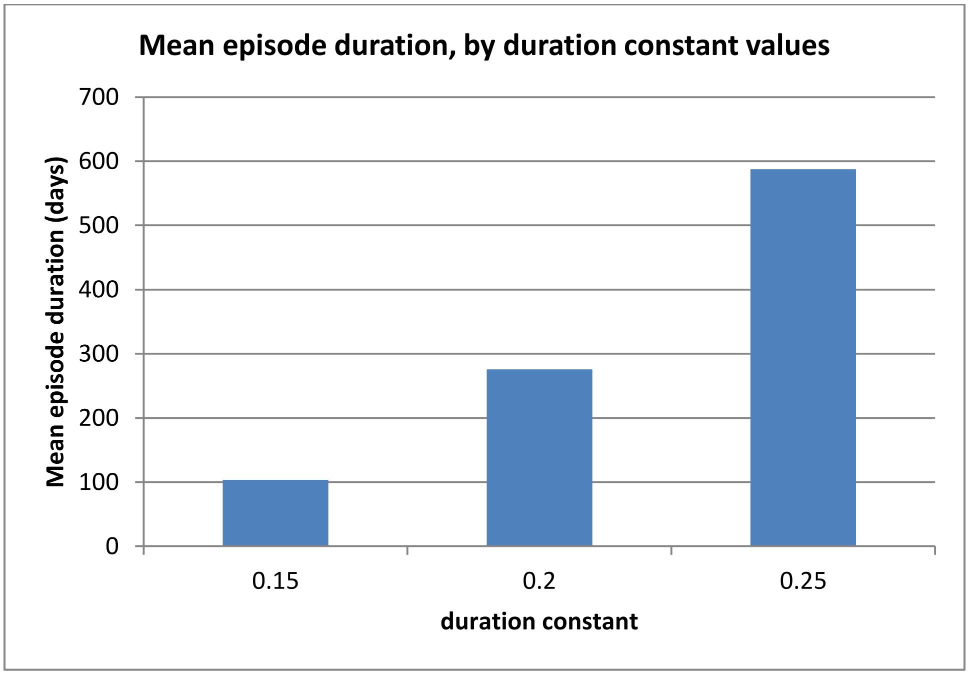

Supplement: Additional file 6 — Model output: Effect of model parameters on incidence and first episode duration. (PDF 297 kb) [file 1471-244X-13-19-S6.pdf]
